# Golden Farina Cologne

**Published:** 1874-09

**Authors:** 


GOLDEN FARINA COLOGNE.—There are thousands of cologne formulas, but the following is said by the *Druggists' Circular* to be superior to most of them:

| | |
|---|---|
| Tincture of Canada snake-root, . . | 4 ounces. |
| Tincture of orris root, . . . | 12 ounces. |
| Oil of bergamot, | |
| Oil of lavender, | |
| Oil of lemon, of each, . . . . | 6 drachms. |
| Essence of musk, | |
| Oil of neroli, | |
| Oil of Cinnamon, | |
| Oil of Cloves, of each, . . . . | 1 drachm. |
| Orange-flower water, . . . . | 8 ounces. |
| Cologne-spirits, sufficient to complete, . | 6 pints. |

